# Relationship between Yes-Associated Protein 1 and Prognosis of Digestive System Neoplasm: Quantitative Analysis and Bioinformatics Analysis Based on 4023 Patients

**DOI:** 10.1155/2022/3798694

**Published:** 2022-07-19

**Authors:** Luo Yuan Yuan, Wu Shuai, Zou Yuan-Peng, Jiang Ting, Xia Wei, He Yu-Qin

**Affiliations:** ^1^Department of Gastroenterology, Daping Hospital, Army Medical University, Chongqing 400042, China; ^2^Key Laboratory of Biorheological Science and Technology, Ministry of Education, College of Bioengineering, Chongqing University, Chongqing 400030, China; ^3^Department of Gastroenterological Surgery, Yong Chuan Hospital of Chongqing Medical University, Chongqing 400016, China; ^4^The College of Foreign Languages and Cultures, Chongqing University, Chongqing 400030, China

## Abstract

Yes-associated protein 1 (YAP1) is involved in the development of a variety of malignancies. However, the prognosis of malignant digestive tumors with YAP1 expression is still controversial. This study searched 31 articles with 36 data sets of 4023 patients to explore the role of YAP1 expression on the prognosis of digestive malignant tumors by searching the PubMed, Embase, Web of Science, Google Scholar, and Cochrane Library databases. Specifically, relevant cancer expression matrix data were downloaded from The Cancer Genome Atlas (TCGA) database. In this meta-analysis, quantitative analysis showed that the overexpression of YAP1 was not conducive to OS (1.62, 95% CI (1.38, 1.90), *P*=0.001) and DFS (1.59, 95% CI (1.31, 1.93), *P*=0.001) in patients with digestive malignant tumors. In addition, TCGA database analysis showed that YAP1 was overexpressed in gastric cancer, cholangiocarcinoma, and colorectal cancer. Survival analysis showed that the patients with high expression of YAP1 in pancreatic cancer have a poor OS (MST: 394 vs. 691 days, *P* < 0.0001) and DFS (MST: 371 vs. 542 days, *P*=0.026) prognosis. YAP1 may be a molecular marker that effectively predicts the survival of malignant digestive tumors, especially pancreatic cancer, and is a potential therapeutic target for malignant digestive tumors.

## 1. Introduction

The global incidence of malignant tumors is increasing year by year. Digestive system malignancies account for about 50% of all malignant tumors, of which gastric cancer (GC), esophageal cancer (EC), liver cancer (HC), and colorectal cancer (CRC) are the most common digestive cancers [1–4]. With the continuous improvement of endoscopic, surgical, chemoradiation, and other treatment methods, the prognosis of patients with malignant digestive tumors has greatly improved, but the overall prognosis is still poor, and the mortality rate remains high. Therefore, it is necessary for us to explore the potential biomarkers and therapeutic targets for digestive malignant tumors and the pathogenesis of malignant tumors, including activation of proto-oncogenes, inactivation of tumor suppressor genes, abnormalities of apoptosis-regulating genes and DNA repair genes, etc.

Yes-associated protein 1 (YAP1) is a proline-rich phosphoprotein [5], located at 11 q22 with a molecular weight of 65,000 Da. The YAP1 protein is an effector molecule of the Hippo pathway. YAP1 is phosphorylated through a phosphorylation cascade [4]. After interacting with 14-3-3, the phosphorylated YAP1 is isolated in the cytoplasm to eliminate biological effects; unphosphorylated Yap1 accumulates in the nucleus and is combined with TEAD domain to induce cell proliferation and differentiation, thereby regulating tissue and organ growth [6, 7]. At the same time, it interacts with Wnt, TGF-*β*, Notch, and other signaling pathways to regulate cell physiological and pathological processes [8]. The Hippo signaling pathway acts as a tumor suppressor pathway. Once activated, its downstream component YAP1 is activated, leading to tumorigenesis and development. Reports showed that the YAP1 gene is amplified in tumors such as esophageal squamous cell carcinoma, medulloblastoma, and liver cancer, which promotes tumorigenesis [9–11]. Bora Singhal et al. [12] have shown that in non-small cell carcinomas, YAP1 binds to Oct4 through the WW domain, inducing Sox2 activation and conferring stem cell-like properties. Zheng et al. [13] showed that Ser127 of YAP1 is the most important phosphorylation site, which determines the subcellular localization of YAP1. Dobutamine attenuates yes-associated protein (YAP)-dependent transcription by inhibiting nuclear translocation of YAP, causing cells to block at G1/S and increase apoptosis. Da et al. [14] have shown that the positive expression of YAP1 is closely related to the clinical stage, tumor size, and lymph node metastasis of gastric cancer. YAP1 may be a prognostic marker of tumors in the digestive system. Due to different test methods, sample content, population, and statistical methods, there are some differences in the results of each study. This article conducts a meta-analysis of previous related studies to resolve the current research controversy. In addition, we also performed a verification analysis through The Cancer Genome Atlas (TCGA) database to further confirm the relationship between YAP1 and the prognosis of malignant digestive tumors.

## 2. Materials and Methods

### 2.1. Retrieval Strategy

By searching the PubMed, Embase, Web of Science, Google Scholar, and Cochrane Library databases and collected the literature on the expression of YAP1 protein on the prognosis of malignant tumors of the digestive system published on February 28, 2020. The English search terms are “Yes-Associated Protein 1,” “YAP1,” “cancer or carcinoma,” “prognosis or prognostic,” and “survival.”

### 2.2. Inclusion and Exclusion Criteria

Inclusion criteria: (1) research on malignant tumors of the digestive system (including the esophagus, stomach, small intestine, colorectum, liver, gallbladder, bile duct, and pancreas); (2) specify the quantitative detection method of YAP1, and clearly explain the high expression of YAP1 or define criteria for low expression; (3) relevant research on YAP1 expression and overall survival (OS) and disease-free survival (DFS); (4) direct or indirect access to hazard ratio (HR) and 95% confidence interval (CI) studies; and (5) detailed information on the study population, study area, and follow-up time obtained. Exclusion criteria: (1) non-digestive system tumors; (2) reviews, reports, and incompletely published studies; (3) repeated publication, repeated inclusion, or similar research; and (4) incomplete data, and the required data unobtainable through calculation.

### 2.3. Data Extraction

Data were extracted by two researchers (Yuan Yuan Luo and Yu-Qin He) in accordance with the principle of independence and differences by discussion. The data included in the study mainly include the name of the first author, the date of publication of the literature, the nationality, tumor type, sample size, YAP1 detection method, cutoff criteria for the high or low expression of YAP1, staining location, type of survival analysis, and hazard ratio (HR) and its 95% CI.

### 2.4. Literature Quality Evaluation

According to the Newcastle-Ottawa Scale (NOS) document quality evaluation scale [15], the quality of the included studies was evaluated independently from three aspects: selection of the study population, comparability, and measurement of the research results. Evaluation was based on the total score of 9 points, and literature scoring ≥7 points was evaluated as high-quality articles.

### 2.5. Statistical Analysis

Stata 14.0 statistical software was used for analysis. Engauge Digitizer 4.1 software was used to extract the survival rate to obtain HR and 95% CI from the original literature that did not directly give HR and 95% CI but only the KM survival curve. The correlation between YAP1 expression and the prognosis of malignant tumors of the digestive system was evaluated by the effect of HR and 95% CI. A meta-analysis was performed on the HR and 95% CI of each study to draw a forest map. Homogeneity tests were performed on the included studies to calculate *I*^2^ statistics to assess heterogeneity between studies. If heterogeneity existed between studies, further subgroup analysis would be performed. Begg's test and Egger's test were used to estimate publication bias. Sensitivity analysis was used to assess the robustness of the combined results. *P* < 0.05 is considered statistically significant.

### 2.6. TCGA Database Analysis

Relevant cancer expression matrix data were downloaded from the TCGA database. The FPKM data format was converted to TPM format, and then normalization (*Z*-score) was performed to extract cancer and adjacent cancer YAP1 gene expression data, as well as clinical follow-up information for each cancer patient. The difference of YAP1 expression between cancer and adjacent cancer was analyzed. The first 25% of YAP1 expression was considered as high expression based on which the relationship between YAP1 and patient prognosis was analyzed.

## 3. Results

### 3.1. Results of Literature Screening

**Figure 1:**
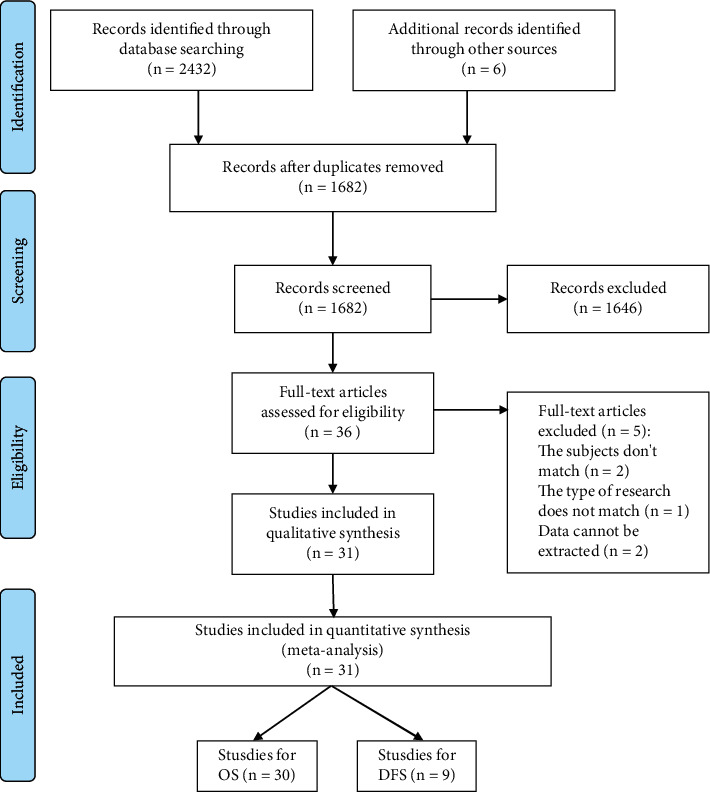
Flowchart of selection process.

A total of 2438 articles were obtained; duplicate articles being excluded. After reading the abstract and the full text and screening the articles according to the inclusion and exclusion criteria (Figure 1), we included a total of 31 [16–46] articles.

### 3.2. The Basic Characteristics of Inclusion in the Literature

A total of 31 articles [16–46] were included, with 36 data sets of involving 4023 patients. There are 30 articles on OS [16–25, 27–46] and 9 articles on DFS [16, 19, 26, 33, 36, 40–42, 46]. One study [36] was carried out in Belgium, six studies [19, 23–25, 33, 43] in Korea, three studies in Japan [18, 32, 35], and twenty-one in China [16, 19–22, 26–31, 34, 37–42, 44–46]. There are 2 articles on pancreatic cancer [35, 42], 7 articles on liver cancer [17, 18, 25, 26, 28, 36, 41], 8 articles on gastric cancer [19–21, 24, 27, 33, 34, 45], 3 articles on esophageal cancer [16, 35, 43], 3 articles on cholangiocarcinoma/gallbladder cancer [18, 26, 28], and 8 articles on colorectal cancer [23, 29, 31, 37, 38, 40, 42, 45]. Two studies used PCR, and 29 studies used IHC. Different studies used different cutoff values. The NOS scores of the included literature were 7–9, all of which were high-quality literature.

### 3.3. Quantitative Synthesis of Analysis Results

**Figure 2:**
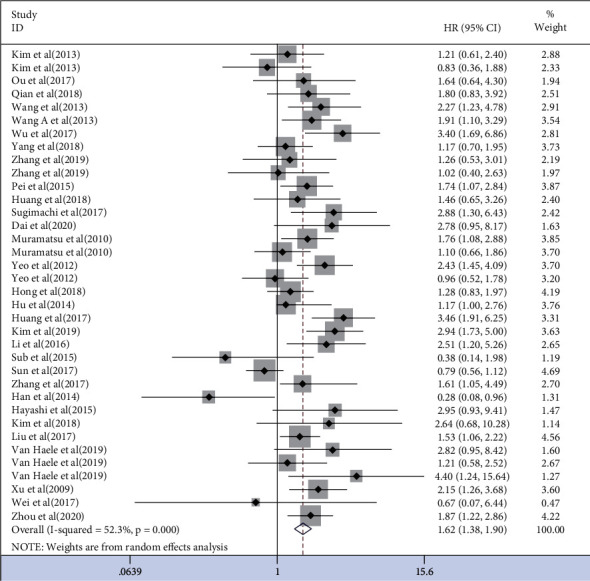
Forest plot of HRs for the association of YAP1 expression with OS.

OS was heterogeneous in 30 studies [12–21, 23–42] (*I*^2^ = 52.3%, *P*=0.001), and a random effects model was used. The results showed that overexpression of YAP1 leads to decreased OS (HR = 1.62, 95% CI (1.38, 1.90), *P*=0.001) (Figure 2).

**Figure 3:**
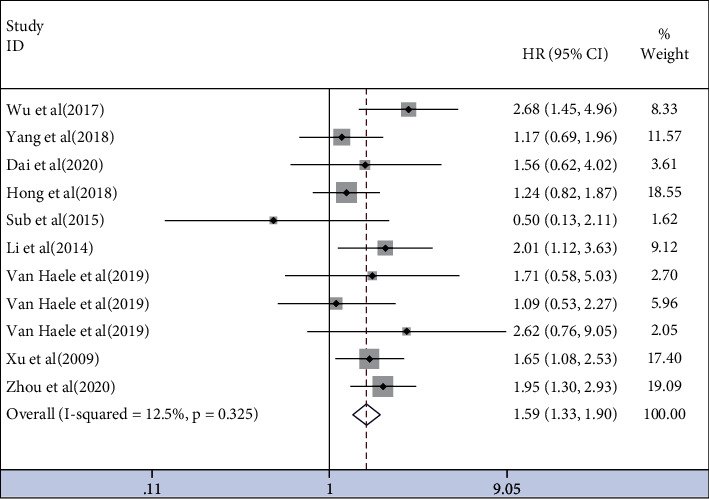
Forest plot of HRs for the association of YAP1 expression with DFS.

Nine studies [16, 19, 26, 33, 36, 40–42, 46] evaluated DFS without heterogeneity (*I*^2^ = 12.5%, *P*=0.325). The results showed that YAP1 overexpression was associated with poor DFS (HR = 1.59, 95% CI (1.31, 1.93), *P*=0.001) (Figure 3).

### 3.4. Subgroup Analysis

Due to the heterogeneity of the OS, we performed a subgroup analysis of the possible factors (tumor type, ethnicity, method, and staining location), as shown in Table 1. The results show that the overexpression of YAP1 acts as a factor leading to poor prognostic in colorectal cancer (HR = 1.56, 95% CI (1.21, 2.02), *P*=0.001), gallbladder carcinoma (HR = 1.87, 95% CI (1.29, 2.71), *P*=0.001), esophageal cancer (HR = 1.58, 95% CI (1.07, 2.32), *P*=0.020), liver cancer (HR = 1.75, 95% CI (1.15, 2.66), *P*=0.009), and pancreatic cancer (HR = 1.81, 95% CI (1.19, 2.74), *P*=0.006); however, there was no effect in gastric cancer (HR = 1.53, 95% CI (0.98, 2.38), *P*=0.059). And then, analyzed by ethnicity, the high expression of YAP1 was associated with poor prognosis in the Asian population (HR = 1.59, 95% CI (1.35, 1.88), *P*=0.001), but not related to non-Asian population (HR = 2.16, 95% CI (0.99, 4.74), *P*=0.554). The subgroup analysis based on the detection method of YAP1 found that significant correlation was observed regardless of whether IHC (HR = 1.59, 95% CI (1.35, 1.87), *P*=0.001) or PCR (HR = 1.62, 95% CI (1.38, 1.90), *P*=0.009) was used. After the sub-localization analysis of YAP1 stained cells, YAP1 expression, nuclear YAP1 expression, and YAP1 mRNA expression were significantly different in tumor patients with poor prognosis (YAP1 expression: HR = 1.63, 95% CI (1.32, 2.02), *P*=0.001; nuclear YAP1 expression: HR = 1.87, 95% CI (1.45, 2.42), *P*=0.001; YAP1 mRNA expression: HR = 2.95, 95% CI (0.93, 9.38), *P*=0.001).

Based on the tumor type, ethnicity, method, and staining location, we performed a subgroup analysis of the studies that reported DFS. As to the tumor type, we found that YAP1 high expression was associated with poor DFS in liver cancer (HR = 1.67, 95% CI (1.25, 2.23), *P*=0.001) and pancreatic cancer (HR = 1.95, 95% CI (1.30, 2.93), *P*=0.001), while colorectal cancer (HR = 1.74, 95% CI (0.77, 3.92), *P*=0.180), esophageal cancer (HR = 1.56, 95% CI (0.61, 3.97), *P*=0.351), and gastric cancer (HR = 1.02, 95% CI (0.49, 2.12), *P*=0.968) have no statistical difference. The subgroup analysis by ethnicity found that high expression of YAP1 was associated with poor DFS in Asian populations (HR = 1.61, 95% CI (1.27, 2.02), *P*=0.001), while there was no statistical difference observed in non-Asian population (HR = 1.45, 95% CI (0.84, 2.49), *P*=0.184). The subgroup analysis based on the YAP1 detection method revealed that IHC (HR = 1.59, 95% CI (1.28, 1.97), *P*=0.001) has found significant correlation, while PCR (HR = 1.56, 95% CI (0.61, 3.97), *P*=0.351) was not statistically significant.

According to the results of YAP1 staining cell sub-localization analysis, YAP1 high expression was associated with the poor prognosis of tumor patients (HR = 1.61, 95% CI (1.30, 1.99), *P*=0.001), while nuclear YAP1 expression (HR = 1.09, 95% CI (0.53, 2.26), *P*=0.816) and cytoplasmic YAP1 expression (HR = 2.62, 95% CI (0.76, 9.04), *P*=0.127) were not statistically significant. The pooled HR for DFS of patients with the expression of YAP1 according to subgroup analyses is shown in Table 2.

### 3.5. Sensitivity Analysis

Sensitivity analysis was performed using the elimination method one by one to explore the impact of a single study on the whole. The results show that the results of this study are stable and unaffected by individual studies (Figures s1A and s2B).

### 3.6. Publication Bias

Begg's test and Egger's test were performed to estimate publication bias. It was found that there was a publication bias in OS (Begg's test: *P*=0.012; Egger's test: *P*=0.005), but there was no publication bias in DFS (Begg's test: *P*=0.497; Egger's test: *P*=0.477) (Figures s2A and s2B).

### 3.7. Results of TCGA Database Analysis

**Figure 4:**
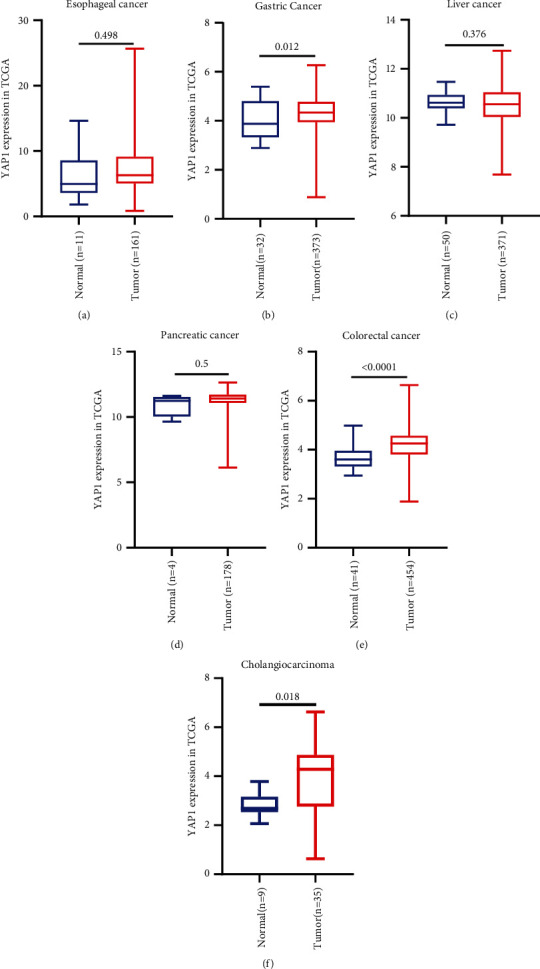
TCGA data analysis of YAP1 expression in tumors of digestive system relative to normal tissues. Expression of YAP1 in esophageal cancer (a), gastric cancer (b), cholangiocarcinoma (c), liver cancer (d), pancreatic cancer (e), and colorectal cancer (f).

**Figure 5:**
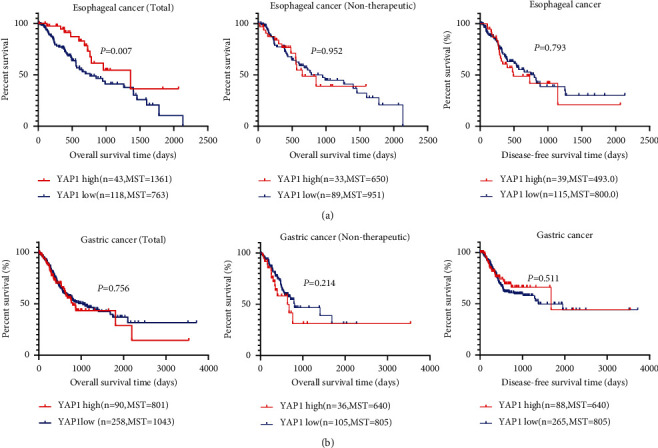
TCGA data analysis the relation of YAP1 expression and prognosis of esophageal cancer (a) and gastric cancer (b).

**Figure 6:**
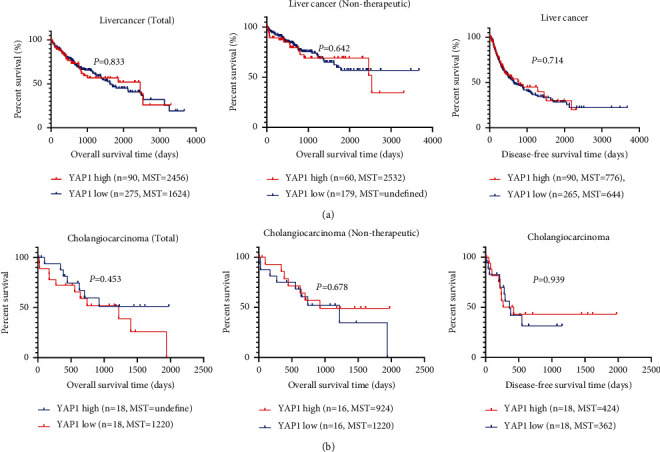
TCGA data analysis the relation of YAP1 expression and prognosis of liver cancer (a) and cholangiocarcinoma (b).

**Figure 7:**
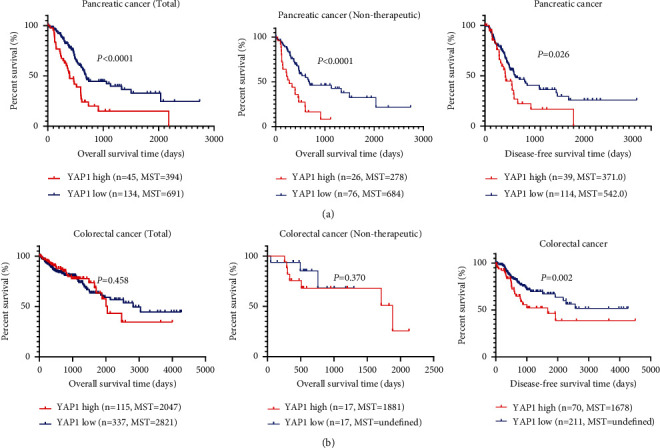
TCGA data analysis the relation of YAP1 expression and prognosis of pancreatic cancer (a) and colorectal cancer (b).

Our analysis of the TCGA database shows that YAP1 is highly expressed in esophageal cancer (*P*=0.498), gastric cancer (*P*=0.012), cholangiocarcinoma (*P*=0.018), pancreatic cancer (*P*=0.018), and colorectal cancer (*P* < 0.0001) relative to normal tissues. The YAP1 expression was not changed in liver cancer (*P*=0.376), but only gastric cancer, cholangiocarcinoma, and colorectal cancer had statistical differences (Figure 4). The survival analysis showed that the relationship between YAP1 expression and overall survival in pancreatic and gastric cancer is consistent with our meta-analysis results (Figures 5–7). Patients with the high expression of YAP1 in pancreatic cancer have a poor prognosis than those with the low expression (MST: 394 vs. 691 days, *P* < 0.0001) (Figure 7(a)); YAP1 expression has no significant effect on overall survival in gastric cancer (MST: 801 vs. 1043 days, *P*=0.756) (Figure 5(b)). Other types of tumors such as cholangiocarcinoma, pancreatic cancer, colorectal cancer, and liver cancer have no statistically significant difference in overall survival time. However, when YAP1 is overexpressed, patients without liver cancer and cholangiocarcinoma have longer median survival time, while those with other types of tumors have a shorter median survival time (Figures 5 and 7). Although there is no statistical difference, the trend is consistent with our meta-analysis results. The overall survival time of patients with overexpression of YAP1 in esophageal cancer is longer than those with low expression, which is contrary to our results of meta-analysis (MST: 1361 vs. 763 days, *P* < 0.0001) (Figure 5). By excluding patients undergoing chemotherapy or radiation, the data showed that the YAP1 expression influences overall survival only in pancreatic cancer (MST: 278 vs. 684 days, *P* < 0.0001) (Figure 7(a)). By the analysis of DFS, we only found that the difference in YAP1 expression affects the median time of DFS in pancreatic cancer (MST: 371 vs. 542 days, *P*=0.026) and colorectal cancer (*P*=0.002) (Figure 7).

## 4. Discussion

One of the important characteristics of malignant tumors is the activation of oncogenes and the inactivation of tumor suppressor genes, which can lead to cancer cell proliferation and promote tumor progression [47, 48]. YAP1 is an oncogene, which mainly promotes abnormal cell proliferation by affecting the expression of cyclins. It also plays an important role in inhibiting apoptosis, loss of cell contact inhibition, and malignant transformation of cells [49, 50]. YAP1 is the main effector downstream of the Hippo signaling pathway. It is a multifunctional intracellular connexin and transcription coactivator, which can have effects on many aspects of human development, growth, DNA repair, and endogenous homeostasis [51, 52]. The abnormal expression of YAP1 is associated with the proliferation and invasion of various tumor cells [53, 54]. Many studies [53, 54] reported the relationship between YAP1 expression and prognosis of gastrointestinal malignant tumors, but the results are not completely consistent.

In this study, we included 31 articles [16–46], with 36 data sets of 4023 patients and performed a quantitative analysis. It was found that the high expression of YAP1 in malignant tumors of the digestive system is closely related to poor prognosis. In addition, we performed a sensitivity analysis and found that the results of this study are stable. And then, a subgroup analysis was performed according to tumor type, ethnicity, method, and staining location. The results showed that the high expression of YAP1 was closely related to the poor OS of Asian population, colorectal cancer, gallbladder carcinoma, esophageal cancer, liver cancer, and pancreatic cancer. High expression is associated with poor DFS in Asian population, liver cancer, and pancreatic cancer.

In order to further verify our findings, we used the TCGA database to analyze the expression of YAP1 in digestive system tumors. YAP1 was elevated in gastric cancer, cholangiocarcinoma, pancreatic cancer, and colorectal cancer. This result is consistent with some previous research. Then, we analyzed the correlation between YAP1 expression and prognosis of patients with digestive system tumors. The YAP1 expression is closely related to prognosis in patients with esophageal and pancreatic cancer. However, YAP1 overexpression in esophageal cancer has a longer median survival time, contrary to some previous research results [12, 31] and our meta-analysis results. So, considering that there may be treatment differences affecting the analysis results, we excluded patients receiving chemoradiotherapy from analysis and found that there was no statistical difference between the YAP1 expression and prognosis in esophageal cancer. This result shows that radiotherapy or chemotherapy is more effective for patients with esophageal cancer with the high expression of YAP1. Regardless of whether the pancreatic cancer is treated or not, the median survival time of patients with the high expression of YAP1 is shorter than that of patients with low expression. There was no statistical difference in the effect of YAP1 expression on overall survival in other types of tumors. Except for liver cancer, when YAP1 is overexpressed, the median survival time is longer. The YAP1 overexpression in other types of tumors has a shorter median survival time than the low expression. Although there is no statistical difference, the trend is consistent with our meta-analysis results. Our analysis of DFS shows that the difference in YAP1 expression is closely related to DFS. The median survival time of patients with high YAP1 expression is shorter than that of patients with colorectal and pancreatic Cancer, and the difference is statistically significant, and the difference is not statistically significant in patients with other types of tumor. The median DFS of patients with the high expression of YAP1 in liver cancer and cholangiocarcinoma is longer, contrary to our results. The median DFS was shorter in patients with high YAP1 expression in esophageal and gastric cancer, consistent with our results.

The differences between this study and the TCGA database may come from the following reasons: first, the detection method is the main reason for this difference. In our quantitative analysis research, IHC was used to detect the expression of the YAP1 protein, while the TCGA database comes from the results of RNA sequencing; and second, due to the difference in treatment, the small number of samples also affects the results.

### 4.1. Limitations

First, it should be emphasized that the heterogeneity of this article cannot be ignored. We cannot change the influence of environmental factors, such as socioeconomic status, follow-up time, and postoperative treatment, which will affect the prognosis. Second, the definition criteria of positive YAP1 expression are not the same. Third, publication bias is another possible cause, and YAP1 is not a predictive factor and may not be published well. The included studies are almost from Asian countries, of which 6 are in Korea [15, 19–21, 29, 39], 3 from Japan [18, 32, 35], and 21 from China [16, 19–22, 26–31, 34, 37–42, 44–46]. Studies of other races have not been found, and this may lead to incomplete race coverage; therefore, it is unknown whether the conclusion can guide other ethnic groups. Finally, since some original studies only provide figures, the data extracted through indirect methods may have some effect on the final results.

## 5. Conclusion

Based on the results of this study, we speculate that YAP1 is a cancer-promoting gene, which is highly expressed in malignant tumors of the digestive system and is closely related to poor prognosis. YAP1 is expected to become a new target for the treatment of malignant tumors of the digestive system. In summary, our research system meta-analyzed the relationship between the expression of YAP1 and the prognosis of digestive system tumors. The overall trend is that the poor prognosis of patients is closely related to the high expression of YAP1. The expression of YAP1 can more accurately predict the prognosis of patients with pancreatic cancer. Therefore, YAP1 may be an effective predictor of digestion molecular markers for surviving systemic malignancies, especially pancreatic cancer, which can provide a new target for the treatment of digestive system tumors such as pancreatic cancer.

## Figures and Tables

**Table 1:** Pooled HR for OS of patients with the expression of YAP1 according to subgroup analyses.

Analysis	No. of studies	HR (95% CI)	*P* value	Heterogeneity	
				*I * ^2^ (%)	*P*
OS	30	1.62 (1.38, 1.90)	0.001	52.3%	0.001
Tumor type					
Colorectal cancer	8	1.56 (1.21, 2.02)	0.001	22.3%	0.238
Cholangiocarcinoma/gallbladder cancer	3	1.87 (1.29, 2.71)	0.001	0.0%	0.457
Esophageal cancer	3	1.58 (1.07, 2.32)	0.020	51.7%	0.082
Gastric cancer	8	1.53 (0.98, 2.38)	0.059	79.3%	0.001
Liver cancer	6	1.75 (1.15, 2.66)	0.009	50.8%	0.047
Pancreatic cancer	2	1.81 (1.19, 2.74)	0.006	0.0%	0.382
Ethnicity					
Asian	29	1.59 (1.35, 1.88)	0.001	53.8%	0.001
Non-Asian	1	2.16 (0.99, 4.74)	0.054	45.0%	0.162
Method					
IHC	28	1.59 (1.35, 1.87)	0.001	53.6%	0.001
PCR	2	1.62 (1.38, 1.90)	0.009	0.0%	0.941
Staining location					
Total YAP1 expression	20	1.63 (1.32, 2.02)	0.001	59.7%	0.001
Nuclear YAP1 expression	9	1.87 (1.45, 2.42)	0.001	19.2%	0.272
Cytoplasmic YAP1 expression	5	1.12 (0.76, 1.65)	0.558	23.9%	0.262
YAP1 mRNA expression	1	2.95 (0.93, 9.38)	0.001	NA	NA

Abbreviations: CI, confidence interval; HR, hazard ratio; IHC, immunohistochemistry; NA, no applicable; OS, overall survival; PCR, polymerase chain reaction.

**Table 2:** Pooled HR for DFS of patients with the expression of YAP1 according to subgroup analyses.

Analysis	No. of studies	HR (95% CI)	*P* value	Heterogeneity	
				*I * ^2^ (%)	*P*
DFS	9	1.59 (1.31, 1.93)	0.001	12.5%	0.325
Tumor type					
Colorectal cancer	2	1.74 (0.77, 3.92)	0.180	75.3%	0.044
Esophageal cancer	1	1.56 (0.61, 3.97)	0.351	NA	NA
Gastric cancer	2	1.02 (0.49, 2.12)	0.968	33.4%	0.221
Liver cancer	3	1.67 (1.25, 2.23)	0.001	0.0%	0.696
Pancreatic cancer	1	1.95 (1.30, 2.93)	0.001	NA	NA
Ethnicity					
Asian	8	1.61 (1.27, 2.02)	0.001	28.1%	0.204
Non-Asian	1	1.45 (0.84, 2.49)	0.184	0.0%	0.459
Method					
IHC	8	1.59 (1.28, 1.97)	0.001	21.2%	21.2%
PCR	1	1.56 (0.61, 3.97)	0.351	NA	NA
Staining location					
Total YAP1 expression	9	1.61 (1.30, 1.99)	0.001	18.0%	0.283
Nuclear YAP1 expression	1	1.09 (0.53, 2.26)	0.816	NA	NA
Cytoplasmic YAP1 expression	1	2.62 (0.76, 9.04)	0.127	NA	NA

Abbreviations: CI, confidence interval; HR, hazard ratio; IHC, immunohistochemistry; NA, no applicable; DFS, disease-free survival; PCR, polymerase chain reaction.

## Data Availability

All data generated or analyzed during this study are included within the article.

## Supplementary Materials

Supplementary Materials Figure s1. A: Sensitivity analysis for all eligible studies investigating the association between YAP1 expression and OS. B: Sensitivity analysis for all eligible studies investigating the association between YAP1 expression and DFS. Figure s2. A: Begg's funnel plot for publication bias test between YAP1 expression and OS. B: Begg's funnel plot for publication bias test between YAP1 expression and DFS.
