# Supplementary material for: Relationship between Yes-Associated Protein 1 and Prognosis of Digestive System Neoplasm: Quantitative Analysis and Bioinformatics Analysis Based on 4023 Patients
Source: Evid Based Complement Alternat Med. 2022 Jul 19;2022:3798694. doi: 10.1155/2022/3798694 (PMC9325623; doi:10.1155/2022/3798694)
Supplement: Supplementary Materials — Figure s1. A: Sensitivity analysis for all eligible studies investigating the association between YAP1 expression and OS. B: Sensitivity analysis for all eligible studies investigating the association between YAP1 expression and DFS. Figure s2. A: Begg's funnel plot for publication bias test between YAP1 expression and OS. B: Begg's funnel plot for publication bias test between YAP1 expression and DFS. [file 3798694.f1.docx]

**Supplementary figure legend**

Figure s1. A: Sensitivity analysis for all eligible studies investigating the association between YAP1 expression and OS. B: Sensitivity analysis for all eligible studies investigating the association between YAP1 expression and DFS.

Figure s2. A: Begg’s funnel plot for publication bias test between YAP1 expression and OS. B: Begg’s funnel plot for publication bias test between YAP1 expression and DFS.

1. Figure s1:

A

B

1. Figure s2:

A

B
